# Management of acetabular fractures in the geriatric patient

**DOI:** 10.1051/sicotj/2017026

**Published:** 2017-05-25

**Authors:** Marc Hanschen, Sebastian Pesch, Stefan Huber-Wagner, Peter Biberthaler

**Affiliations:** 1 Department of Trauma Surgery, Klinikum rechts der Isar, Technical University Munich Ismaninger Strasse 22 81675 Munich Germany

**Keywords:** Acetabular fracture, Elderly patient, Primary total hip replacement, Individualized treatment, Patient risk factors, Injury risk factors

## Abstract

*Introduction*: Open reduction and internal fixation (ORIF) is standard care for most acetabular fractures. With increasing numbers of acetabular fractures in the elderly, the risk of revision surgery and conversion to total hip replacement (THR) is increasing. Alarmingly, about 20–25% of acetabular fractures in the elderly following ORIF needed revision and conversion to delayed THR.

*Methods*: Recently, prognostic factors have been identified, which correlate with an increased risk of worse outcomes following ORIF of acetabular fractures in the elderly patient. Patient risk factors include, for example, age, comorbidities, and degree of osteoporosis. Injury risk factors mainly include the fracture pattern.

*Results*: The concept of primary THR following acetabular fractures is an alternative to ORIF, especially in the elderly patient. Satisfactory outcomes have been reported in different studies for primary THR following acetabular fractures in the elderly. The surgeon should be aware of strict selection criteria in order to achieve these satisfactory outcomes. Therefore, an individualized treatment plan has to be defined for elderly patients following acetabular fractures.

*Discussion*: Here, the advantages and disadvantages of ORIF versus THR following acetabular fractures in the elderly are discussed.

## Introduction

The overall incidence of fractures of the acetabulum has, despite introduction of higher safety standards in cars, not undergone significant changes. The incidence ranges at about three patients/100,000/year [1]. The occurrence is bimodal, i.e. we observe two age peaks of patients suffering from acetabular fractures. Young patients suffer from acetabular fractures due to high energy trauma (e.g. motor vehicle accident, sporting injury, etc.), this has to be distinguished from elderly patients suffering from low energy trauma (e.g. drops, falls). Interestingly, which is most likely to be demographically driven, the mean age of patients suffering from acetabular fractures is increasing [1, 2]. A contributing factor seems to be the more active lifestyle in the elderly population [3].

With the increasing age, treatment of patients following acetabular fractures becomes more challenging. Not only comorbidities with its subsequent increase in perioperative complications, but also the poor bone quality (osteoporosis) tends to result in unfavorable outcomes [4]. Several prognostic factors have been identified to determine the outcome. They can be categorized as patient factors, injury factors, and treatment factors [4]. Of these three entities, only treatment factors can be influenced in order to determine the patient’s outcome following acetabular fractures. Patient factors include age, degree of osteoporosis, comorbidities, presence of degenerative joint disease, premorbid activity level, and mental function [4]. Injury factors include the presence of associate injuries and the fracture pattern [4, 5]. In elderly patients, especially comminuted fractures of the posterior wall or the quadrilateral surface have been identified to be prognostic factors determining functional outcome. Further prognostic injury factors include the degree of intra-articular comminution, articular cartilage damage, and the location and degree of femoral head impaction. In addition, the timing of presentation has to be considered, delayed presentation is associated with unfavorable outcomes. Treatment factors include the management chosen (conservative, open reduction and internal fixation, total hip arthroplasty acute or delayed), the management of perioperative complications, and the quality and timing of the named management [4].

Aiming to create and maintain concentric reduction of the joint line and avoiding secondary operation, open reduction and internal fixation (ORIF) has been recognized as the treatment of choice for displaced acetabulum fractures [6]. Due to the above-named patient and injury factors, ORIF is associated with compromised outcomes especially in the elderly patient population. According to a recent systemic review (15 studies included), about 23% of patients older than 55 years underwent revision with conversion to total hip arthroplasty following fixation of acetabular fractures at a mean of 64 months (ORIF and percutaneous fixation) [7]. Due to the increasing number of elderly patients suffering from acetabulum fractures and the increased risk of unfavorable outcomes, it has been recognized to apply individual management approaches taking into account patient and injury factors in order to yield successful outcomes [4, 8].

## Classification and fracture patterns in the geriatric patient

Judet et al. suggested a classification based on the anatomical and biomechanical concepts of the acetabulum to be formed by two columns [9]. The classification distinguishes between five elementary fracture patterns (posterior wall, posterior column, anterior wall, anterior column, transverse) and five associated fracture patterns (posterior wall and column, transverse posterior wall, T-shaped, anterior with posterior hemi-transverse, both columns). The Judet and Letournel classification has been shown to have a high intra- and interobserver reliability, therefore it is widely accepted [10]. Based on this classification, a comprehensive classification of acetabular fractures was developed by SICOT International Documentation and the AO/ASIF Foundation. Based on morphologic characteristics, acetabular fractures are divided into types, groups, and subgroups. The comprehensive classification is especially helpful in research applications.

Both column fractures account for about 20–30% of all fractures, this fracture pattern is observed in young and old patients [7, 11]. Due to the predominant injury mechanism in the elderly being low energy falls and the osteoporotic bone structure, typically lateral compression injuries result [12]. Low energy falls on the greater trochanter result in comminution of the anterior column, anterior wall, and quadrilateral plate. In contrast, younger patients are more likely to suffer from high energy trauma with fractures of the posterior column, posterior wall, and transverse fractures [7, 11, 13]. The combination of high degree of comminution, medial roof impaction, quadrilateral plate fracture, and concomitant injury of the femoral head has been identified to be risk factors for poor outcomes in the elderly following open reduction and internal fixation [14, 15].

## Conservative management

Conservative management of acetabular fractures in the elderly is restricted to a select patient population and select fracture patterns. Moribund patients with significant premorbidities or severely limited preinjury mobility may qualify for conservative management of acetabular fractures [4, 15]. In addition, fractures should only be minimally displaced and intrinsically stable (e.g. anterior column fractures) or exhibit secondary congruence of the hip joint [15]. Even in the seldom cases of iliac pseudarthrosis following conservative management of acetabular fractures, it is the degree of congruency determining the functional outcome of the hip joint [16].

Of note, conservative treatment has to be rather considered conservative management, including sufficient pain control, physiotherapy, deep venous thrombosis (DVT) prophylaxis, close clinical follow-up examinations, and radiologic follow-up examinations. During follow-up examinations, not only late displacement has to be excluded, but also aggravation of premorbidities due to immobility. Early mobilization is mandatory as strict bed rest may result in medical complications associated with prolonged recumbence [15].

Recent findings underline that traction should not be used for the treatment of acetabular fractures in the elderly. First, capsular ligamentotaxis is insufficient in retaining reduction as deforming forces are typically rotational and not translational [15]. Second, traction therapy is associated with medical complications due to prolonged recumbence, additionally pin disengagement and pullout in osteoporotic bones is encountered frequently with subsequent risk of osteomyelitis [17].

Only limited data concerning the expected clinical outcome following conservative management is present in the literature. Along with the high risk of severe complication like DVT due to prolonged recumbence, about 33% of elderly patients treated conservatively following acetabular fractures suffer from poor and unacceptable functional results [18, 19].

### Open reduction and internal fixation (ORIF)

Although outcomes of ORIF following acetabular fractures in the elderly are worse than in the younger population, ORIF remains to be the standard care for most fractures of the acetabulum in the elderly [20, 21]. Predictors for beneficial functional outcomes, in young and old patients, include an anatomic acetabular reduction which is achieved and maintained through healing, a preserved femoral head, and the lack of perioperative complications [4]. In addition, timing of surgery has been identified to be crucial in obtaining favorable outcomes. Due to callus, organizing hematoma, and granulation tissue, the anatomic reduction rate of acetabular fractures decreases significantly following 11 days [22]. Moreover, there is a strong correlation between the functional outcomes following acetabular surgery across all ages between the rate of reduction and the experience of the surgeon, as shown by Matta and coworkers [19]. Stratifying the functional outcome rates by age, at first sight it does not seem to be surprising that poor outcomes strongly correlate with the age of the patient [4, 23]. Increasing age, osteoporosis, and inactivity are associated with a deterioration of functional outcomes [23]. However, further studies, controlling the quality of fracture reduction for the series, revealed that when anatomic reduction could be achieved, the outcomes were comparable in younger and elderly patients [21]. Proper selection of patients, suitable for ORIF following acetabular fractures, is associated with good outcomes, this could be shown by Helfet and Virkus in 2002 (unpublished data). In their study, the overall complication rate was 4–7%. In their study, ORIF was only indicated in patients meeting the following criteria: Fracture pattern reducible and fixable via a single non-extensile approach, adequate bone quality, lack of femoral head injury, no need for trochanteric osteotomy or disruption of abductor muscles, and reasonable surgical time (<3–4 h).

**Figure 1.**
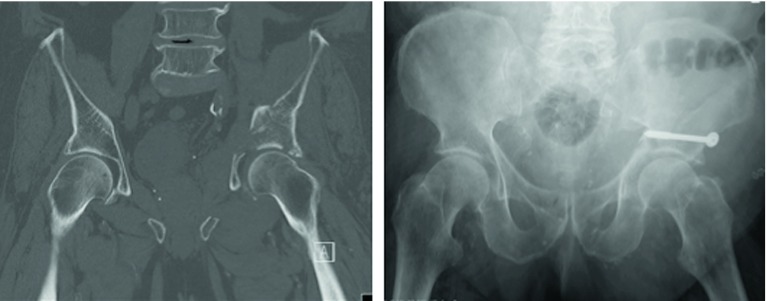
Moribund 81-year-old patient following low energy fall. Besides cardiomyopathy, significant lung fibrosis excluded extensive surgical therapy. The individualized treatment plan was defined as minimal invasive ORIF of the anterior column + posterior hemi-transverse fracture. Minimal invasive surgery was conducted using a cannulated 7.3 mm screw. The postoperative AP view was acquired on the intensive care unit (ICU). No additional follow-up could be conducted, the patient passed away two months following surgery in the rehabilitation unit.

Minimal invasive osteosynthesis following acetabular fractures has been reported earlier [24], accurate reduction is nearly impossible. Due to the fact that anatomical reduction is a strong predictor for the clinical outcome following acetabular fractures, minimal invasive approaches with the placement of cannulated screws should be the exception. Moribund patients might benefit from minimal invasive approaches (Figure 1).

Due to improvements in available osteosynthesis materials, beneficial outcomes in the elderly will most likely be easier to achieve in the future. Angular stable implants and refined anatomical designs, like quadrilateral surface plates to buttress the medial wall of the acetabulum, increase the stability of osteosynthesis especially in osteoporotic bone. Future clinical studies will need to address the potential beneficial effects.

**Figure 2.**
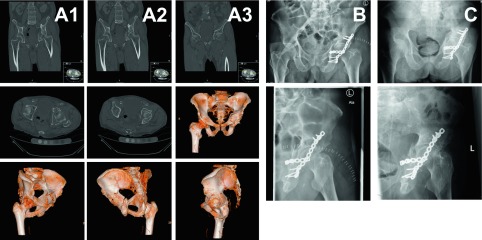
Two-column fracture of the left acetabulum following a bicycle accident in a 76-year-old male. Significant premorbidities included cardiomyopathy with s/p myocardial infarction nine years ago, recurrent DVTs with warfarin anticoagulation, lower limb atherosclerosis with s/p femoro-popliteal bypass, and s/p viral encephalitis with residual impaired gait and presence of suprapubic catheter. Due to s/p multiple abdominal surgeries, the anterior approach was no option. The individualized treatment plan was defined as ORIF via one singular Kocher-Langenbeck approach, aiming to convert the two-column fracture into a stable fracture pattern with secondary congruence of the hip joint. Computed tomography (CT) scans show the two-column fracture in frontal, axial views and 3D reconstruction (column A1–A3), postoperative conventional radiographs display the reduced joint line at two weeks (column B) and at three months follow-up (column C).

Careful analysis of patient factors and injury factors has to be conducted in order to make an informed decision whether ORIF can be applied or not (Figure 2). Besides the above-named prognostic factors the evident or potential injury of the femoral head has to be included into the decision process. Matta and coworkers defined injuries of the femoral head as the most important factor in determining poor outcomes following ORIF in acetabular fractures [23]. Unfortunately, injuries of the femoral head are not always evident in conventional radiographs. Impactions and fractures can be easily missed [25]. Occult fractures or development of avascular necrosis following acetabular fractures are more frequent in elderly patients with impaired bone quality and with comminuted and extended fracture patterns [26]. Taking into account the above-named prognostic factors, associated with a high likelihood of failure following ORIF, is essential in determining the treatment plan in elderly patients following acetabular fractures.

Outcome analysis showed a high rate of failure of ORIF and conversion to Total Hip Replacement (THR). Numbers range between 10% for the general population [27], case series in elderly patients showed significantly higher numbers with up to 30% of conversion to THR [28]. As mentioned above, the outcome following ORIF can be compromised due to insufficient fragment reduction, avascular necrosis, heterotopic ossification, or preexisting osteoarthritis, these cases are associated with the need for revision surgery [23, 29].

## Secondary total hip replacement (THR)

ORIF is considered standard care for most fractures of the acetabulum, including fractures in the elderly [21]. Unfortunately, especially in the elderly, high rates of revisions are observed following ORIF of acetabular fractures after months or years [28]. Treatment of choice in these cases is secondary THR. The belated arthroplasty has to be considered challenging as initial surgery may have induced scar tissue, heterotopic ossifications, soft tissue or bone defects, avascularity, and the possibility of occult infection [25]. In addition, bone stock loss and abnormal anatomy as well as impeding hardware have been associated with an increased failure rate of delayed THR [30]. Therefore, a thorough workup is needed prior to conversion to secondary THR, especially occult infections have to be excluded by a thorough clinical examination and laboratory workup. Extended radiologic workup might be needed, like magnetic resonance imaging (MRI) scanning or arteriography. At the time of surgery, special implants need to be at hand in order to account for bone stock loss or abnormal anatomy situations. Although numerous studies addressed the outcome following delayed THR following acetabular fractures, no clear recommendations can be given. This is due to the heterogeneity of studies present, most studies did not stratify the results according to the injury type, the type of ORIF, nor the age [4]. In addition, the clinical trials present in the current literature reflect treatment techniques and implant choices, which have to be considered outdated by now [4]. Nevertheless, the following seems to apply for secondary THR following acetabular fractures [4]: Delayed THR can provide satisfactory outcomes, bone grafting and reconstruction of structural anatomy is associated with improved results, cemented cups should be used, and secondary THR following ORIF is more demanding than after closed treatment (Table 1). In case of an extensile approach and absence of contraindications, heterotopic ossification prophylaxis should be initiated.

**Table 1.** Primary and Secondary total hip replacement (THR) following acetabular fractures. In order to define the individualized treatment plan for elderly patients with acetabular fractures, advantage and disadvantage of primary and secondary THR have to be considered.

	Total hip replacement (THR) following acetabular fractures
Primary THR following acetabular fractures	Advantages: Good functional outcomes reported. Immediate weight bearing possible.
	Disadvantages: Risk of aseptic loosening. Stable fixation challenging due to fracture situation, comminution, and poor bone quality. Risk of dislocation (esp. in patients with cognitive or neurological impairment). Risk of heterotopic ossifications. Thorough preoperative planning necessary, special implants might be needed (e.g. Müller acetabular reinforcement ring).
Secondary THR following acetabular fractures	Advantages: Defined bone stock, (mostly) consolidated fracture. Bone grafting, cemented cups, and reconstruction of anatomy associated with improved outcomes.
	Disadvantages: Failure rate higher due to abnormal anatomy, impeding hardware, and/or bone stock loss. Thorough preoperative planning necessary, special implants might be needed (e.g. Müller acetabular reinforcement ring). Surgery challenging due to scar tissue, heterotopic ossifications, soft tissue or bone defects, avascularity, and the possibility of occult infection.

The risk of heterotopic ossification is increased in technically difficult cases, with increased surgery duration and local tissue trauma. In addition, early dislocation of the prosthesis and postoperative hematoma are risk factors for heterotopic ossifications [31]. Two prophylactic modalities are accepted, treatment with anti-inflammatory drugs and irradiation. Especially in the elderly population treatment with anti-inflammatory drugs like indomethacin or ibuprofen might be associated with undesirable effects like gastritis, bleeding, or renal damage. Single dose irradiation therapy might be an alternative in patients at risk for undesirable effects of anti-inflammatory medication. Of note, irradiation is associated with impaired fracture healing and higher risk for non-union [32].

## Primary total hip replacement (THR)

In 1954 primary THR was initiated for displaced acetabular fractures [33]. In the following years, clinical trials underlined the role of primary THR following acetabular fractures due to good functional outcomes [34, 35]. Although good functional outcomes have been reported, acute THR following acetabular fractures remains controversial. This is mostly attributed to heterogeneous studies comparing different treatment protocols. In addition, the selection criteria have to be defined strictly for patients to benefit from primary THR. Increased age has been identified to be a prognostic factor for poor outcomes [25]. Furthermore, cognitive and neurological impairment has been identified as risk factor. Due to higher incidences of dislocations in patients with cognitive impairment [36], the use of acute THR is debated in this patient population. The most common complications following acute THR remain secondary dislocations and heterotopic ossifications [37].

**Figure 3.**
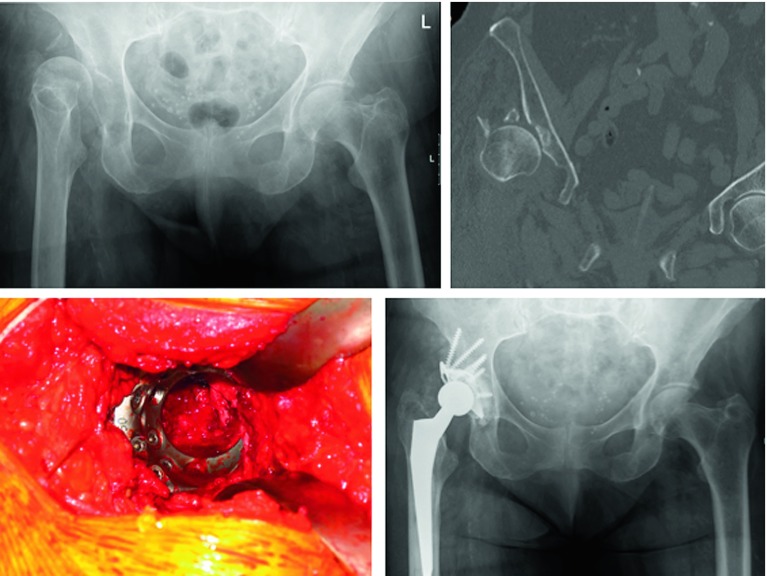
Due to a low energy fall a 84-year-old female patient presented with a Pipkin-IV fracture. The femoral head and the acetabular rim were fractured, the patient presented with a dislocation of the hip. Due to atrial fibrillation permanent anticoagulation with warfarin was present. Following bridging, the individualized treatment included acute total hip replacement (THR) with a Müller acetabular reinforcement ring.

**Figure 4.**
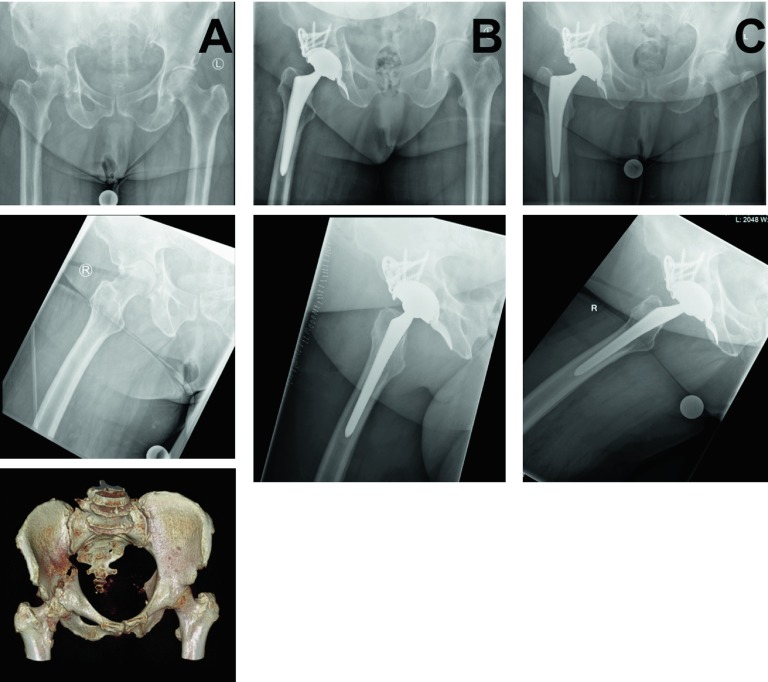
Fracture of the right acetabulum of a 67-year-old female. The patient reported to the emergency room four weeks delayed, upon admission impaction of the femoral head and the pattern of a two-column fracture was visible (column A). Significant comorbidities were missing, the individualized treatment plan was defined as acute total hip replacement (THR) with application of the Burch-Schneider reinforcement ring. Follow-up X-rays at two weeks (column B) and at three months (column C) are shown.

The biggest perioperative challenge in performing acute THR remains to be aseptic loosening following cup insertion [25]. Due to the fracture situation, along with poor bone quality and comminution in the elderly, in order to achieve stable fixation of the cup the surgeon needs to perform a thorough analysis of the fracture pattern and has to be equipped with a thorough understanding of the cup options. Individualized treatment approaches are needed in order to achieve stable fixation of the cup. Among the different treatment options, two main strategies can be applied. First, minimal invasive reduction and fixation of the fracture with acute THR. Second, acute THR without reduction and fixation of the fracture. In the latter case, stable fixation is being achieved using special implants, for example the Müller acetabular reinforcement ring (Figure 3). Clinical studies suggest comparable outcomes applying these two strategies. Acute THR without attempting to reduce the fracture with application of the Burch-Schneider reinforcement ring (Figure 4) and autologous bone grafting of the acetabulum led to good clinical outcomes in a study with 10 patients [38]. The authors reported complete incorporation of the bone graft and no loosening. Comparable results were reported in a study following up on 10 patients with rigid internal fixation and primary THR [39]. At a follow-up of 36 months, all fractures united without signs of loosening. Due to small study populations and inhomogeneous patient populations, especially concerning fracture pattern and comorbidities, the optimal strategy for successful implantation of an acute THR following acetabular fractures remains to be controversially discussed. Individual treatment strategies need to be defined, based on thorough analysis of patient factors, injury factors, and treatment factors available.

## Conclusion

**Figure 5.**
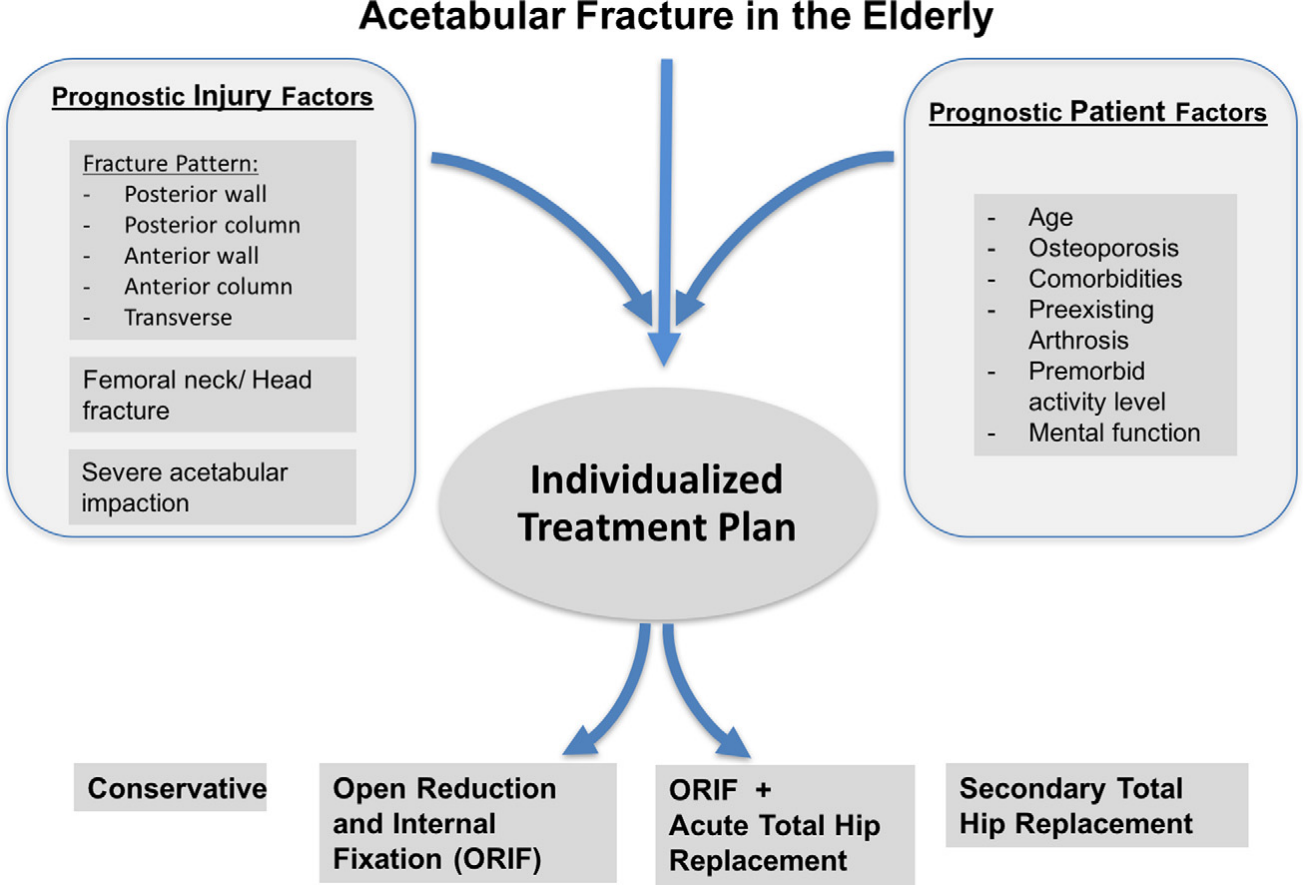
Algorithm for individualized treatment of acetabular fractures in the elderly. Prognostic injury and patient factors have to be considered prior to determining the treatment.

Acetabular fractures in the elderly remain to be a challenge. Patient and injury factors, e.g. age, premorbidities, and certain fracture patterns, have been identified to be prognostic factors for the clinical outcome. Prior to defining the management of these injuries, assessment of these prognostic factors is a prerequisite for successful treatment of acetabular fractures in the elderly. The treatment should be individualized (Figure 5), we strongly recommend to define an individualized treatment plan following acetabular fractures in the elderly taking into account injury factors and patient factors. Conservative treatment remains to be the exception. Although ORIF is considered to be standard care for acetabular fractures, high failure rates have been reported for elderly patients. Acute THR can provide satisfactory outcomes, a prerequisite is a thorough understanding of the fracture pattern, a concise assessment of the patient risk and prognostic factors, and the right choice of implant in the fracture situation.

## Conflict of interest

All authors declare to have no conflict of interest.
